# Supplementary material for: Transfer-RNA-Derived Fragments Are Potential Prognostic Factors in Patients with Squamous Cell Carcinoma of the Head and Neck
Source: Genes (Basel). 2020 Nov 13;11(11):1344. doi: 10.3390/genes11111344 (PMC7698123; doi:10.3390/genes11111344)
Supplement: Supplementary file 1 [file genes-11-01344-s001.zip › supplementary/Figure S1.docx]

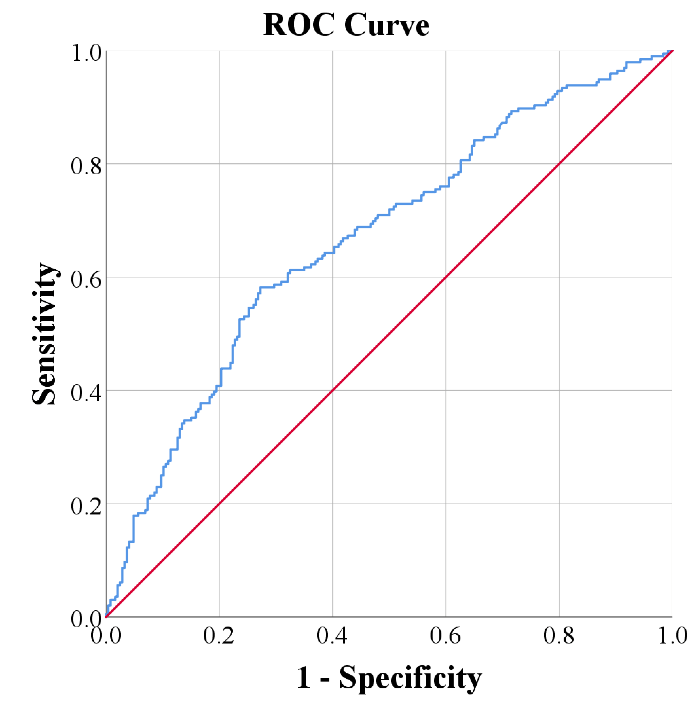


**Figure S1**. ROC curve showing the sensitivity and specificity of tRF-20

in predicting patient overall survival (AUC = 0.669, *p* < 0.001).
