# Supplementary material for: Transfer-RNA-Derived Fragments Are Potential Prognostic Factors in Patients with Squamous Cell Carcinoma of the Head and Neck
Source: Genes (Basel). 2020 Nov 13;11(11):1344. doi: 10.3390/genes11111344 (PMC7698123; doi:10.3390/genes11111344)
Supplement: Supplementary file 1 [file genes-11-01344-s001.zip › supplementary/Table S1 Genes.docx]

Table S1. Clinical data on 12 healthy controls and 16 patients with SCCOT (own clinical samples)

| ID | Sample | Age | Sex | TNM | Clinical Stage | Localization ^#^ | Status ^§^ | Follow-up Months | Months to Recurrence |
| --- | --- | --- | --- | --- | --- | --- | --- | --- | --- |
| NT2 | Healthy controls | 49 | Female |  |  |  |  |  |  |
| NT3 |  | 25 | Female |  |  |  |  |  |  |
| NT4 |  | 30 | Male |  |  |  |  |  |  |
| NT5 |  | 27 | Male |  |  |  |  |  |  |
| NT6 |  | 42 | Female |  |  |  |  |  |  |
| NT7 |  | 32 | Female |  |  |  |  |  |  |
| NT8 |  | 41 | Female |  |  |  |  |  |  |
| NT9 |  | 35 | Female |  |  |  |  |  |  |
| NT10 |  | 57 | Male |  |  |  |  |  |  |
| NT11 |  | 45 | Male |  |  |  |  |  |  |
| NT13 |  | 48 | Female |  |  |  |  |  |  |
| NT14 |  | 59 | Female |  |  |  |  |  |  |
| p35 | Tumor and tumor-free samples | 24 | Female | T2N0M0 | II | 1 | DOD | 13 | 10 |
| p49 |  | 52 | Female | T4N2cM0 | IV | 3 | DWD | 3 |  |
| p56 |  | 40 | Female | T2N2bM0 | IV | 3 | DOD | 16 | 12 |
| p58 |  | 61 | Male | T1N0M0 | I | 1 | ADF | 147 |  |
| p73 |  | 80 | Male | T4aN0M0 | IV | 3 | DOD | 19 | 11 |
| p76 |  | 58 | Male | T4aN0M0 | IV | 3 | DDF | 114 |  |
| p79 |  | 60 | Male | T1N0M0 | I | 2 | ADF | 123 |  |
| p83 |  | 64 | Female | T1N0M0 | I | 2 | ADF | 122 |  |
| p124 |  | 54 | Male | T4aN2bM0 | IV | 3 | DOD | 3 |  |
| p131 |  | 74 | Female | T2N0M0 | II | 2 | ADF | 65 |  |
| p137 |  | 71 | Female | T2N0M0 | II | 2 | ADF | 65 |  |
| p138 |  | 50 | Male | T2N1M0 | III | 2 | ADF | 64 |  |
| p149 |  | 69 | Female | T1N0M0 | I | 2 | DDF | 18 |  |
| p154 |  | 42 | Female | T1N1M0 | III | 1 | ADF | 56 |  |
| p157 |  | 68 | Male | T1N0M0 | I | 3 | ADF | 56 |  |
| p187 |  | 73 | Female | T1N0M0 | I | 1 | ADF | 46 |  |

^#^ Localization: 1, tongue; 2, lateral border of the tongue; 3, tongue with overgrowth outside the mobile tongue. ^§^ Status: DWD, dead with disease; DDF, dead disease-free; ADF, alive disease-free; DOD, dead of disease
