# Supplementary material for: Transfer-RNA-Derived Fragments Are Potential Prognostic Factors in Patients with Squamous Cell Carcinoma of the Head and Neck
Source: Genes (Basel). 2020 Nov 13;11(11):1344. doi: 10.3390/genes11111344 (PMC7698123; doi:10.3390/genes11111344)
Supplement: Supplementary file 1 [file genes-11-01344-s001.zip › supplementary/Table S2 Genes.docx]

Table S2. Genomic locations of the 15 prognostic tRFs in SCCHN

| **Fragment sequence** | **Type** | **MINTbase Unique ID (sequence derived)** | **MINTbase Alternative IDs (GRCh37 assembly-derived) ^#^** | **D-loop overlap?** | **Anticodon-loop overlap?** | **Anticodon-triplet overlap?** | **T-loop overlap?** |
| --- | --- | --- | --- | --- | --- | --- | --- |
| TTTGGGTGCGAGAGGTCCCGGGT | i-tRF | tRF-23-Z87HFK8SDZ | trna6_ProTGG_14_+_21152175_21152246@31.53.23 | no | full | full | partial |
| TTTGGGTGCGAGAGGTCCCGGGT | i-tRF | tRF-23-Z87HFK8SDZ | trna28_ProTGG_16_-_3234133_3234204@31.53.23 | no | full | full | partial |
| TTTGGGTGCGAGAGGTCCCGGGT | i-tRF | tRF-23-Z87HFK8SDZ | trna3_ProTGG_16_+_3208923_3208994@31.53.23 | no | full | full | partial |
| TTTGGGTGCGAGAGGTCCCGGGT | i-tRF | tRF-23-Z87HFK8SDZ | trna8_ProTGG_16_+_3238094_3238165@31.53.23 | no | full | full | partial |
| TTTGGGTGCGAGAGGTCCCGGGT | i-tRF | tRF-23-Z87HFK8SDZ | trna14_ProTGG_5_-_180615854_180615925@31.53.23 | no | full | full | partial |
| TTTGGGTGCGAGAGGTCCCGGGT | i-tRF | tRF-23-Z87HFK8SDZ | trna3_ProTGG_14_+_21101165_21101236@31.53.23 | no | full | full | partial |
| TTTGGGTCCGAGAGGTCCC | i-tRF | tRF-19-Z8SSFKJJ | trna12_ProTGG_11_-_75946869_75946940@31.49.19 | no | full | full | no |
| TGCCGTGATCGTATAGTGGTTAGTACTCTG | 5'-tRF | tRF-30-XSXMSL73VL4Y | trna111_HisGTG_1_-_147774845_147774916@-1T.29.30 | full | no | no | no |
| TGCCGTGATCGTATAGTGGTTAGTACTCTG | 5'-tRF | tRF-30-XSXMSL73VL4Y | trna118_HisGTG_1_-_145396881_145396952@-1T.29.30 | full | no | no | no |
| TGCCGTGATCGTATAGTGGTTAGTACTCTG | 5'-tRF | tRF-30-XSXMSL73VL4Y | trna16_HisGTG_1_+_146544773_146544844@-1T.29.30 | full | no | no | no |
| TGCCGTGATCGTATAGTGGTTAGTACTCTG | 5'-tRF | tRF-30-XSXMSL73VL4Y | trna21_HisGTG_1_+_147753471_147753542@-1T.29.30 | full | no | no | no |
| TGCCGTGATCGTATAGTGGTTAGTACTCTG | 5'-tRF | tRF-30-XSXMSL73VL4Y | trna1_HisGTG_15_+_45493349_45493420@-1T.29.30 | full | no | no | no |
| TGCCGTGATCGTATAGTGGTTAGTACTCTG | 5'-tRF | tRF-30-XSXMSL73VL4Y | trna8_HisGTG_15_-_45492611_45492682@-1T.29.30 | full | no | no | no |
| TGCCGTGATCGTATAGTGGTTAGTACTCTG | 5'-tRF | tRF-30-XSXMSL73VL4Y | trna9_HisGTG_15_-_45490804_45490875@-1T.29.30 | full | no | no | no |
| TGCCGTGATCGTATAGTGGTTAGTACTCTG | 5'-tRF | tRF-30-XSXMSL73VL4Y | trna33_HisGTG_6_+_27125906_27125977@-1T.29.30 | full | no | no | no |
| TGCCGTGATCGTATAGTGGTTAGTACTCTG | 5'-tRF | tRF-30-XSXMSL73VL4Y | trna7_HisGTG_9_-_14433938_14434009@-1T.29.30 | full | no | no | no |
| TCCTAAGCCAGGGATTGTGGGT | i-tRF | tRF-22-8XF6RE98N | trna12_ArgCCT_16_+_3243918_3243990@33.54.22 | no | partial | full | partial |
| TCCTAAGCCAGGGATTGTGGGT | i-tRF | tRF-22-8XF6RE98N | trna3_ArgCCT_7_+_139025446_139025518@33.54.22 | no | partial | full | partial |
| TCCTAAGCCAGGGATTGTGGGT | i-tRF | tRF-22-8XF6RE98N | trna2_ArgCCT_16_+_3202901_3202973@33.54.22 | no | partial | full | partial |
| TCCTAAGCCAGGGATTGTGGGT | i-tRF | tRF-22-8XF6RE98N | trna21_ArgCCT_17_-_73030526_73030598@33.54.22 | no | partial | full | partial |
| TCCTAAGCCAGGGATTGTGGGT | i-tRF | tRF-22-8XF6RE98N | trna18_ArgCCT_17_+_73030001_73030073@33.54.22 | no | partial | full | partial |
| TCCGGCTCGAAGGACC | 3'-tRF | tRF-16-884U1DD | trna19_TyrGTA_14_-_21121258_21121351@60.75.16 | no | no | no | partial |
| TCCGGCTCGAAGGACC | 3'-tRF | tRF-16-884U1DD | trna17_TyrGTA_14_-_21128117_21128210@60.75.16 | no | no | no | partial |
| TCCGGCTCGAAGGACC | 3'-tRF | tRF-16-884U1DD | trna16_TyrGTA_14_-_21131351_21131444@60.75.16 | no | no | no | partial |
| TCCGGCTCGAAGGACC | 3'-tRF | tRF-16-884U1DD | trna5_TyrGTA_14_+_21151432_21151520@60.75.16 | no | no | no | partial |
| TCCGGCTCGAAGGACC | 3'-tRF | tRF-16-884U1DD | trna14_TyrGTA_6_+_26569086_26569176@60.75.16 | no | no | no | partial |
| TCCGGCTCGAAGGACC | 3'-tRF | tRF-16-884U1DD | trna15_TyrGTA_6_+_26575798_26575887@60.75.16 | no | no | no | partial |
| TCCGGCTCGAAGGACC | 3'-tRF | tRF-16-884U1DD | trna2_TyrGTA_2_+_27273650_27273738@60.75.16 | no | no | no | partial |
| TCCGGCTCGAAGGACC | 3'-tRF | tRF-16-884U1DD | trna4_TyrGTA_8_+_67025602_67025694@60.75.16 | no | no | no | partial |
| TCCGGCTCGAAGGACC | 3'-tRF | tRF-16-884U1DD | trna5_TyrGTA_8_+_67026223_67026311@60.75.16 | no | no | no | partial |
| GTTAAAGACTTTTTCTCTGAC | 3'-tRF | tRF-21-7OFIZ9WUD | trnaMT_ProTGG_MT_-_15956_16023@49.69.21 | no | no | no | full |
| GTCTCTGTGGCGCAATGGAC | 5'-tRF | tRF-20-S998LO9D | trna86_ArgTCT_1_-_159111401_159111474@1.20.20 | partial | no | no | no |
| GGCCGGTTAGCTCAGTCGGC | 5'-tRF | tRF-20-6S7P4PWJ | trna57_IleAAT_6_+_27636362_27636435@1.20.20 | partial | no | no | no |
| GCTTCTGTAGTGTAGTGGT | 5'-tRF | tRF-19-Q99P9PJZ | trna152_ValCAC_6_-_27248049_27248121@1.19.19 | partial | no | no | no |
| CTTTGAATCCAGCGATCCGAG | i-tRF | tRF-21-NYDRFU8U0 | trna130_GlnTTG_6_-_27763640_27763711@32.52.21 | no | full | full | no |
| CTTTGAATCCAGCGATCCGAG | i-tRF | tRF-21-NYDRFU8U0 | trna173_GlnTTG_6_-_26311975_26312046@32.52.21 | no | full | full | no |
| CTTTGAATCCAGCGATCCGAG | i-tRF | tRF-21-NYDRFU8U0 | trna174_GlnTTG_6_-_26311424_26311495@32.52.21 | no | full | full | no |
| CTTTGAATCCAGCGATCCGAG | i-tRF | tRF-21-NYDRFU8U0 | trna16_GlnTTG_17_+_47269890_47269961@32.52.21 | no | full | full | no |
| CGGATAGCTCAGTCGGTAGA | i-tRF | tRF-20-MEF91SS2 | trna5_LysTTT_11_+_59323902_59323974@4.23.20 | full | no | no | no |
| CGGATAGCTCAGTCGGTAGA | i-tRF | tRF-20-MEF91SS2 | trna54_LysTTT_1_+_204475655_204475727@4.23.20 | full | no | no | no |
| CGGATAGCTCAGTCGGTAGA | i-tRF | tRF-20-MEF91SS2 | trna62_LysTTT_1_-_204476158_204476230@4.23.20 | full | no | no | no |
| CGGATAGCTCAGTCGGTAGA | i-tRF | tRF-20-MEF91SS2 | trna14_LysTTT_11_-_59327808_59327880@4.23.20 | full | no | no | no |
| CGGATAGCTCAGTCGGTAGA | i-tRF | tRF-20-MEF91SS2 | trna2_LysTTT_17_+_8022473_8022545@4.23.20 | full | no | no | no |
| CGGATAGCTCAGTCGGTAGA | i-tRF | tRF-20-MEF91SS2 | trna76_LysTTT_6_+_28918806_28918878@4.23.20 | full | no | no | no |
| CGAATCCGGCTCGAAGGACCA | 3'-tRF | tRF-21-LE3JWB61B | trna14_TyrGTA_6_+_26569086_26569176@56.76.21 | no | no | no | partial |
| CGAATCCGGCTCGAAGGACCA | 3'-tRF | tRF-21-LE3JWB61B | trna15_TyrGTA_6_+_26575798_26575887@56.76.21 | no | no | no | partial |
| ATTGGTCGTGGTTGTAGTCCG | i-tRF | tRF-21-I8W47W1R0 | trnaMT_GluTTC_MT_-_14674_14742@39.59.21 | no | no | no | full |
| ATTGGTCGTGGTTGTAGTCCG | i-tRF | tRF-21-I8W47W1R0 | trnalookalike8_GluTTC_5_-_93905172_93905240@39.59.21 | no | no | no | full |
| ATTGGTCGTGGTTGTA | i-tRF | tRF-16-I8W47WB | trnaMT_GluTTC_MT_-_14674_14742@39.54.16 | no | no | no | partial |
| ATTGGTCGTGGTTGTA | i-tRF | tRF-16-I8W47WB | trnalookalike8_GluTTC_5_-_93905172_93905240@39.54.16 | no | no | no | partial |
| ATAGTGGTTAGTACTCTGCGTTG | i-tRF | tRF-23-H3RXSINH0P | trna111_HisGTG_1_-_147774845_147774916@12.34.23 | full | partial | partial | no |
| ATAGTGGTTAGTACTCTGCGTTG | i-tRF | tRF-23-H3RXSINH0P | trna118_HisGTG_1_-_145396881_145396952@12.34.23 | full | partial | partial | no |
| ATAGTGGTTAGTACTCTGCGTTG | i-tRF | tRF-23-H3RXSINH0P | trna16_HisGTG_1_+_146544773_146544844@12.34.23 | full | partial | partial | no |
| ATAGTGGTTAGTACTCTGCGTTG | i-tRF | tRF-23-H3RXSINH0P | trna21_HisGTG_1_+_147753471_147753542@12.34.23 | full | partial | partial | no |
| ATAGTGGTTAGTACTCTGCGTTG | i-tRF | tRF-23-H3RXSINH0P | trna1_HisGTG_15_+_45493349_45493420@12.34.23 | full | partial | partial | no |
| ATAGTGGTTAGTACTCTGCGTTG | i-tRF | tRF-23-H3RXSINH0P | trna8_HisGTG_15_-_45492611_45492682@12.34.23 | full | partial | partial | no |
| ATAGTGGTTAGTACTCTGCGTTG | i-tRF | tRF-23-H3RXSINH0P | trna9_HisGTG_15_-_45490804_45490875@12.34.23 | full | partial | partial | no |
| ATAGTGGTTAGTACTCTGCGTTG | i-tRF | tRF-23-H3RXSINH0P | trna33_HisGTG_6_+_27125906_27125977@12.34.23 | full | partial | partial | no |
| ATAGTGGTTAGTACTCTGCGTTG | i-tRF | tRF-23-H3RXSINH0P | trna7_HisGTG_9_-_14433938_14434009@12.34.23 | full | partial | partial | no |

^#^ tRNA number_Amino acid and anticodon_Chromosome_Strand_Start position_End position@Start position relative to start of mature tRNA.End position relative to start of mature tRNA.Fragment length
