# Supplementary material for: Transfer-RNA-Derived Fragments Are Potential Prognostic Factors in Patients with Squamous Cell Carcinoma of the Head and Neck
Source: Genes (Basel). 2020 Nov 13;11(11):1344. doi: 10.3390/genes11111344 (PMC7698123; doi:10.3390/genes11111344)
Supplement: Supplementary file 1 [file genes-11-01344-s001.zip › supplementary/Table S3 Genes.docx]

Table S3. Clinical variables and their associations with tRF-20 (TCGA, SCCHN)

|  | | **tRF-20 level**  **(log2-transformed RPM)** | | | ***p*-value** |
| --- | --- | --- | --- | --- | --- |
|  |  | **< 2** | **2 - 4** | **> 4** |  |
| **Gender** | **Female** | 38 | 51 | 41 | 0.713 |
|  | **Male** | 104 | 115 | 94 |  |
| **Age (years)** | **<= 40** | 4 | 8 | 5 | 0.107 |
|  | **41 - 65** | 87 | 111 | 72 |  |
|  | **> 65** | 50 | 47 | 58 |  |
| **HPV infection** | **Negative** | 115 | 129 | 94 | 0.869 |
|  | **Positive** | 23 | 26 | 16 |  |
| **Clinical stage** | **1** | 4 | 8 | 6 | 0.625 |
|  | **2** | 51 | 62 | 58 |  |
|  | **4** | 84 | 90 | 69 |  |
