# Supplementary material for: Transfer-RNA-Derived Fragments Are Potential Prognostic Factors in Patients with Squamous Cell Carcinoma of the Head and Neck
Source: Genes (Basel). 2020 Nov 13;11(11):1344. doi: 10.3390/genes11111344 (PMC7698123; doi:10.3390/genes11111344)
Supplement: Supplementary file 1 [file genes-11-01344-s001.zip › supplementary/Table S4 Genes.docx]

Table S4. tRF-20 levels (RPM) in different SCCHN subtypes

| **Site of resection or biopsy** | **N** | **Median** | **Minimum** | **Maximum** |
| --- | --- | --- | --- | --- |
| Tongue, NOS | 113 | 6.989 | 1.024 | 171.893 |
| Larynx, NOS | 99 | 7.193 | 1.074 | 307.970 |
| Overlapping lesion of lip, oral cavity and pharynx | 64 | 14.688 | 1.110 | 177.923 |
| Floor of mouth, | 46 | 4.982 | 1.888 | 96.283 |
| Tonsil, NOS | 27 | 5.923 | 1.066 | 101.652 |
| Mouth, NOS | 22 | 7.187 | 1.003 | 160.020 |
| Base of tongue, NOS | 19 | 5.633 | 1.164 | 134.770 |
| Cheek mucosa | 14 | 6.160 | 1.816 | 35.760 |
| Gum, NOS | 8 | 7.652 | 1.631 | 64.298 |
| Hypopharynx, NOS | 7 | 9.378 | 2.269 | 171.226 |
| Oropharynx, NOS | 6 | 11.431 | 1.437 | 67.679 |
| Hard palate | 3 | 19.367 | 3.281 | 29.357 |
| Lip, NOS | 3 | 2.502 | 1.149 | 9.939 |
| Anterior floor of mouth | 2 |  | 1.637 | 9.715 |
| Lower gum | 2 |  | 1.392 | 2.093 |
| Mandible | 1 |  | 3.531 | 3.531 |
| Palate, NOS | 1 |  | 114.163 | 114.163 |
| Pharynx, NOS | 1 |  | 2.013 | 2.013 |
| Posterior wall of oropharynx | 1 |  | 5.403 | 5.403 |
| Retromolar area | 1 |  | 2.306 | 2.306 |
| Supraglottis | 1 |  | 1.964 | 1.964 |
| Upper gum | 1 |  | 1.377 | 1.377 |
| Total | 442 | 7.511 | 1.003 | 307.970 |
